# Age-Related Differences in Physical Fitness and Performance of an “Ability Test” among Firefighters

**DOI:** 10.3390/muscles3010009

**Published:** 2024-03-07

**Authors:** Koulla Parpa, Marcos Michaelides

**Affiliations:** Faculty of Sports and Exercise Science, UCLan University of Cyprus, Avenue 12-14, 7080 Pyla, Cyprus; mmichaelides@uclan.ac.uk

**Keywords:** simulated firefighting tasks, ability test, body composition, occupational, abdominal muscles, upper body muscular endurance

## Abstract

This study’s primary objective was to examine the differences in body composition, abdominal strength, absolute and relative power, handgrip strength, one repetition maximum for squat and bench press, and the maximum count of push-up and sit-up repetitions executed within a minute across different age cohorts of firefighters. Furthermore, this study aimed to evaluate the age-related differences in firefighters’ completion times of six firefighting tasks. Eighty-four male volunteer firefighters (age 33.79 ± 6.97 years) were grouped into three age categories, 20–30 years, 31–40 years, and 41–50 years, and underwent the aforementioned evaluations. One-way analysis of variance (MANOVA) revealed that age exerts a statistically significant influence (*p* < 0.001) on body fat percentage, waist circumference, and waist-to-hip ratio. Furthermore, age significantly affected the overall time of the ability test (*p* < 0.001) and the duration required to accomplish each individual task (*p* < 0.001). Additionally, age significantly affected abdominal strength, relative power (as measured by the step test), and the maximum count of push-up and sit-up repetitions performed within a minute. These outcomes support earlier research indicating an age-associated decrement in physical fitness parameters among firefighters. It is recommended that firefighters prioritize maintaining strength and endurance of the abdominal muscles, upper body muscular endurance, and a healthy body weight. The emphasis on specific muscular groups is essential for improving task performance within this profession.

## 1. Introduction

Firefighting is widely recognized as a profession that imposes substantial physical and psychological challenges [1,2,3]. The capacity of firefighters to safely fulfil their duties in diverse environments is closely linked to their health status and their ability to attain superior levels of physical fitness [4,5]. Firefighters frequently engage in activities such as climbing ladders and stairs and manipulating fully charged hoses, all while wearing substantial protective clothing and self-contained breathing apparatus (SCBA) [6]. These activities increase physiological demands and maximal heart rates, potentially leading to cardiovascular strain [6]. Research affirms that for firefighters to manage those job stressors and reduce the possibility of musculoskeletal injuries, they should maintain high levels of cardiovascular health, muscular strength, and endurance [7,8]. Concurrently, research indicates that performance of occupational tasks is positively associated with abdominal strength, upper body endurance (as measured by push-ups), upper body strength (via one repetition of max bench press), and power (as assessed by a step test) [9,10]. Conversely, poor performance of firefighting tasks is associated with high resting heart rates, high body fat percentage [11], high body mass index, large waist, and increasing age [9,10].

Maintaining high levels of physical fitness, which is considered crucial to performing occupational tasks effectively, can be challenging, particularly with advanced age. Research indicated that the risk of health complaints among older Dutch firefighters was six times higher than that of their younger colleagues [12]. Studies conducted on firefighters revealed a decline in the aerobic capacity of older firefighters when compared to the minimum required threshold [13] and a lower work performance, especially in those over 50 years [14]. Low cardiorespiratory fitness has been identified as a contributory factor towards the onset of health problems such as obesity, hypertension, and heart disease, all of which are increasing with advanced age [15]. Moreover, research conducted by Ras et al. (2023) investigating the relationship between physical fitness and cardiovascular well-being in firefighters revealed that poor cardiovascular health was negatively associated with relative oxygen consumption, leg strength, and the count of push-up repetitions [16]. In addition, age was inversely related to maximal oxygen consumption, number of push-ups, sit-up repetitions, and flexibility [16]. Concurrently, a negative correlation was observed between body fat percentage and absolute maximal oxygen consumption, leg strength, handgrip strength, the count of push-up and sit-up repetitions performed, and lean body mass [16]. Comparable results were documented in a study of Spanish firefighters, which identified age, weight, and total body fat as significant predictors of maximal oxygen consumption, with total body fat being identified as the most significant predictor [17].

A reduction in physical fitness with ageing is expected, considering that advanced age is accompanied by changes in the musculoskeletal system, including a reduction in skeletal muscle mass and bone strength and an increase in body fat [18]. These negative physiological changes, in addition to the changes in the cardiovascular system [13,16] with advanced age, may be translated into reductions in physical fitness and work capacity [14]. Researchers propose that firefighters who maintain elevated levels of physical activity and healthy body weight are more likely to maintain better cardiorespiratory fitness needed to perform their occupational activities safely [19]. Similarly, Bennet and Colleagues (2011) suggested that maintaining cardiovascular fitness, which is essential for meeting the high physical demands of firefighting, appears to be influenced more by measures of overweight/obesity and self-reported regular activity than age in firefighters up to 58 years [20]. Conversely, the findings of Saari and Colleagues (2020) revealed that physically trained firefighters in the older age group exhibited lower physical capabilities in comparison to their younger peers, even though they reported comparable training routines [21].

Although a considerable number of studies have explored the impact of age on firefighting capabilities, further investigation is needed to assess the influence of age on various fitness parameters that have been identified to contribute to firefighting performance [9,10]. Moreover, exploring the influence of age on an ability test that incorporates simulated firefighting activities is necessary. Despite the apparent role of anaerobic capacity in firefighting tasks, investigations devoted to anaerobic power and anaerobic capacity are limited. Unlike aerobic capacity, which maximal oxygen consumption tests can easily evaluate, there is no single test to assess anaerobic capacity. Thus, anaerobic power in this study was evaluated through the anaerobic step test (AST) and vertical jump. This study is unique because testing was part of the firefighters’ assessment; therefore, all the city firefighters participated in the evaluations, irrespective of their rank or fitness level. Consequently, the goal of the present study was to explore differences in anthropometric and body composition measures, abdominal strength, absolute and relative power (as assessed by vertical jump and step test), handgrip strength, the one repetition maximum for squat and bench press, and the maximum number of push-up and sit-up repetitions performed within a minute among firefighters across various age groups. Additionally, this study aimed to assess the age differences in firefighters’ completion times of six firefighting tasks. We hypothesized that significant differences in body composition, abdominal strength, absolute and relative power, handgrip strength, one repetition maximum for squat and bench press, and the maximum count of push-up and sit-up repetitions would be observed among firefighters of different age groups, with the older firefighters demonstrating significantly reduced performance compared to their younger counterparts.

## 2. Materials and Methods 

### 2.1. Study Design

This research was conducted in two stages: initially, the firefighters performed the ability test (AT), and approximately two weeks later, they underwent the fitness assessments. Firefighting performance was evaluated through the AT, which the fire department designed to assess firefighters’ performance under simulated scenarios. Therefore, during the test, all firefighters were required to wear protective gear weighing 22.68 kg to simulate actual firefighting demands. The cumulative time to conclude the test, as well as the specific durations to accomplish each firefighting task, was recorded. The test was administered at the main fire station, engaging firefighters across all three shifts. The administration of the test was overseen by the same researchers, assisted by firefighter instructors (who were trained in the appropriate techniques and procedures), and comprised six timed tasks. The environmental conditions were recorded and monitored during the sessions, with the average temperature and humidity being 10.56 C (November) and 35%, respectively. Fitness testing was carried out approximately two weeks later, following the same strategy. All the firefighters were familiar with the testing as this was part of their regular assessment. Prior to the initiation of testing, each firefighter received a briefing on the procedures and provided their informed consent. The study adhered to ethical standards in alignment with the Declaration of Helsinki and received approval from the University’s Research Review Board.

### 2.2. Participants

Ninety male volunteer firefighters took part in the study (33 ± 7 years). Due to incomplete measurements, eighty-four were included in this study (age 33.79 ± 6.97 years, height 181.19 ± 6.44 cm, body weight 96.94 ± 15.20 kg). For the study, participants were grouped into three age categories: 20–30 years (n = 30), 31–40 years (n = 37), and 41–50 years (n = 17).

### 2.3. Ability Test (AT) Procedures 

Tasks were presented in the sequence of their execution. The total time and the individual times for each task were recorded. 

The initial task (*stair climb*) required participants to engage in both ascending and descending a staircase composed of twelve steps (each step measuring 24 × 30.5 cm). The activity was to be completed a total of eight times.

The second task (*lift and move the rolled hose*) entailed the transportation of six hoses over a distance of 4.1 m. Each hose rolled for the task measured was 15.24 m in length and 7 in diameter with a weight of 9.53 kg. Participants were required to lift the hoses individually from the ground to the bench. Upon placing all rolls on the bench, participants then stepped back from the bench and proceeded to return the rolls to the ground in the same configuration as initially presented. The firefighters needed to ensure the rolls were stacked neatly, with explicit instruction to avoid dropping or tossing the hoses.

The third task (*Keiser sled*) required propelling a 68.8 kg I-beam across a distance of 1.5 m on a Keiser Sled employing a 4.1 sledgehammer. Participants were directed to execute over-the-head swings to strike the I-beam. 

During the fourth task (*hose pull and hydrant hookup*), participants were required to position themselves within a 2 × 2 m square and manually pull an uncharged (dry) fire hose, 7 cm in diameter, across a distance of 31.5 m. Following the retraction of the hose into the square, they needed to hook the hose to the hydrant. This involved manually unscrewing the cap from the hydrant and securely fastening a hose coupling onto it. The task ended as they were instructed to remove the coupling by hand and replace it with the small cap that had been removed from the hydrant. 

The fifth task (*rescue mannequin drag*) entailed dragging an 82 kg mannequin over a distance of 15.7 m using both hands. Participants needed to approach the mannequin from the rear, lift it from the shoulders, and proceed to drag it by walking in reverse. 

During the sixth task (*charged hose advance*), participants were tasked with lifting a nozzle attached to a charged hose (4.4 cm) and advancing the hose line over a distance of 15.24 m. Once the nozzle passed the finish line, the test was completed. The overall duration of the test was also recorded. 

### 2.4. Anthropometric Measurements

A wall stadiometer (Leicester; Tanita, Tokyo, Japan) was utilized to determine participants’ stature, and a bioelectrical impedance analyzer (BF3222; Tanita) was used to assess body composition. Prior to the bioelectrical impedance testing, the firefighters were directed to adhere to the established protocols [22], and the testing was conducted following the manufacturer’s manual. According to the guidelines, hip and waist measurements were obtained twice and recorded to the nearest 0.1 cm [23]. 

### 2.5. Muscular Endurance

The one-minute sit-up test was utilized to evaluate the muscular endurance of the abdominal region with the count of accurately executed sit-ups in sixty seconds. The test–retest reliability for this assessment has been shown to vary between r = 0.68 and r = 0.91 [24]. To evaluate the muscular endurance of the upper body, the maximum push-up test was utilized, with its test test–retest reliability reported as r = 0.93 [24]. 

### 2.6. Strength

The one repetition maximum (1-RM) tests for bench press and squat were conducted to measure upper and lower body strength, respectively, adhering to the established protocols [25]. Upper body strength was evaluated using a handgrip dynamometer (TKK 5401, Takei Scientific Instruments, Ltd., Tokyo, Japan), with the test–retest reliability of the test reported as r = 0.90 [24]. Abdominal strength was measured using the ABMED isometric device (Abdominal Measuring Device S and B Associates Ltd., Springdale, AR, USA) following the standardized guidelines [26]. Firefighters were positioned on an incline bench, with arms crossed over the chest and heels secured on a footrest, to exert maximum force against a cushioned arm linked to a force gauge. The peak force output, displayed on a gauge meter (Omega DP41-S, Norwalk, CT, USA) after three attempts, was recorded for analysis. The correlation coefficient was high between the three trials (r values between 0.97 and 0.98, *p* < 0.001).

### 2.7. Anaerobic Power Testing 

The anaerobic step test (AST) was performed in line with the established guidelines [23], calculating power (W) using the formula [body weight in kg × 10] × 0.40 × number of steps × 1.33])/time in seconds. Here, 0.40 represents the step height (40 cm), and 1.33 is the factor for the eccentric portion of the exercise, translating the equation into total work. The duration of the test time was 60 s, with its test–retest reliability reported as high (r = 0.90) [23]. Jumping performance was measured with the vertek device (Sports Imports, Columbus, Worthington, OH, USA) following the specific guidelines [23]. The participants performed three jumps, and the best one was recorded for further analysis. Power was determined by the formula: power (kg.m^−1^ s^−1^) = 2.21 × body weight (in kg) × square root of jumping height.

#### Statistical Analysis

Statistical analyses were conducted using IBM^®^ SPSS^®^ Statistics, version 28.0 (SPSS Inc., Chicago, IL, USA). All data are reported as mean values and standard deviations (SDs) following confirmation of normal distribution. A one-way multivariate analysis of variance (MANOVA) was conducted to identify the differences in firefighting and fitness parameters between the three age groups. When differences between the three age groups were identified, the analysis was followed by pairwise comparisons. The magnitude of the differences was quantified using a partial eta-squared (η^2^) measure. To provide meaningful analysis for comparisons between groups, eta-squared effect sizes were considered small for values between 0.1 and 0.5, medium for values up to 1.3, and large for values over 1.4 [27]. Significance was set at *p* < 0.05.

## 3. Results

Table 1 displays the anthropometric and body composition data. The results from the one-way multivariate analysis of variance (MANOVA) revealed significant age-related differences in these measurements (F = 19.52, *p* = 0.00, Wilk’s Λ = 0.10, partial η^2^ = 0.69). Further examination showed that age significantly influences body fat percentage [F (2, 77) = 6.37, *p* = 0.003, partial η^2^ = 0.14)], waist circumference [F (2, 77) = 3.23, *p* = 0.043, partial η^2^ = 0.08)], and waist-to-hip ratio [F (2, 77) = 4.99, *p* = 0.01, partial η^2^ = 0.12)]. However, no significant age-related differences were found for body weight, body mass index (BMI), or hip circumference.

Table 2 outlines the firefighters’ completion times. The results revealed significant variations in both overall and specific task completion times by age (F = 2.59, *p* = 0.00, Wilk’s Λ = 0.65, partial η^2^ = 0.19). Further analysis showed a significant impact of age on the overall time to complete the ability test [F (2, 81) = 11.58, *p* = 0.00, partial η^2^ = 0.22)], stair climb [F (2, 81) = 9.45, *p* = 0.00, partial η^2^ = 0.19)], rolled hose lift and move [F (2, 81) = 13.63, *p* = 0.00, partial η^2^ = 0.25)], Keiser sled [F (2, 81) = 6.20, *p* = 0.00, partial η^2^ = 0.13)], rescue mannequin drag [F (2, 81) = 4.78, *p* = 0.01, partial η^2^ = 0.11)], and charged hose advance [F (2, 81) = 5.15, *p* = 0.01, partial η^2^ = 0.11)]. Age did not significantly affect the completion time for the hose pull and hydrant hookup. Detailed differences between the age groups are presented in Table 2.

Firefighters’ performance of physical fitness tests is presented in Table 3. The results indicated age-related differences in firefighters’ outcomes on physical fitness assessments (F = 2.04, *p* = 0.01, Wilk’s Λ = 0.56, partial η^2^ = 0.25). Specifically, age significantly influenced abdominal strength [F (2, 77) = 3.54, *p* = 0.03, partial η^2^ = 0.08)], relative power on the step test [F (2, 77) = 6.24, *p* = 0.00, partial η^2^ = 0.14)], the maximum count of push-ups [F (2, 77) = 5.04, *p* = 0.01, partial η^2^ = 0.12)], and the number of sits-ups completed within a minute [F (2, 77) = 5.48, *p* = 0.01, partial η^2^ = 0.13)]. Conversely, age was not found to significantly affect vertical jump’s relative or absolute power, handgrip strength, 1-RM bench press, 1-RM squat, or the step test’s absolute power. 

## 4. Discussion

The results of this investigation contribute to the existing knowledge by demonstrating that age has a statistically significant impact on body fat percentage, waist circumference, and waist-to-hip ratio. Conversely, no statistical differences were observed in terms of body weight, BMI, or hip circumference as a function of age based on our results. Further analyses revealed that age significantly influences the total duration required to complete the ability test, including specific activities such as stair climbing, the rolled hose lift and move, the Keiser sled, rescue mannequin drag, and the advance with a charged hose. However, age did not appear to significantly affect the times for hose pull and hydrant hookup. Moreover, this study found that age significantly affects abdominal strength, relative power during the step test, and the maximum count of push-up and sit-up repetitions performed within a minute. In contrast, no significant age-related differences were detected in the relative or absolute power of the vertical jump, handgrip strength, one-repetition maximum (1-RM) for bench press, 1-RM for squat, or the absolute power exhibited during the step test. 

Our research findings revealed a significant increase in the percentage of body fat among firefighters aged 41 and older, with a mean value of 26.13 ± 5.59. Nonetheless, there were no significant differences in body fat percentage observed among the younger age cohorts, with averages of 20.40 ± 4.72 for those aged 20–30 years and 22.62 ± 5.61 for those aged 31–40 years. The same was true for the waist-to-hip ratio, where the firefighters aged 41 and above had significantly greater ratios (0.95 ± 0.05) compared to the younger groups (0.88 ± 0.07 and 0.90 ± 0.07 for 20–30 and 31–40 years, respectively), even though no significant differences were identified in the hip circumferences. In our study, body fat measurements for the 41–50 age group were greater than those reported by previous studies and similar to the levels reported in firefighters aged over 55 [17]. In contrast, the measurements for the younger groups closely aligned with those reported by earlier investigators studying Spanish firefighters [17] but were greater than those reported in a study that involved individuals registered as competitors at the world championship Scott Firefighter Combat Challenge course [21]. The variations in body fat percentage observed between our findings and those of other studies may be due to the characteristics of the sample being studied, such as participation in different wellness programs [28], differences in rank [29], etc., or variations in the assessment methods [28]. A recent study conducted over a five-year period in the United States, which investigated weight changes among firefighters aged below 45 years and those aged 45 years and above, revealed obesity rates of 25% among the younger group and 35% among the older group [30]. Notably, younger firefighters experienced a significantly higher weight gain compared to their older counterparts [30]. Moreover, a meta-analysis demonstrated that the prevalence rates of overweight and obesity within the firefighter population were 44.1% and 35.6%, respectively [31]. In parallel, the prevalence rate of abdominal obesity among firefighters was documented at 37.9% [31]. Last but not least, Mayer and Colleagues (2012) explored the relationship between obesity and the endurance of back and abdominal muscles [32]. The authors indicated that there were significant negative correlations between BMI and body fat percentage with the endurance of back and core muscles. The regression analyses conducted by the authors revealed that BMI, body fat percentage, as well as age and self-reported physical activity levels explained 17–19% of the variability in back muscular endurance and 29–37% of the variability in core muscular endurance [32]. 

Subsequent analysis revealed that age had a statistically significant impact on the total duration needed to complete the various components of the ability test. This includes climbing stairs, lifting and moving the rolled hose and Keiser sled, dragging a rescue mannequin, and advancing a charged hose. However, age did not significantly impact the times for hose pulling and hydrant hookup tasks. Our results are in agreement with previously published studies, which demonstrated that the time to complete simulated tasks tends to decline with age [33]. Davis et al. (1982) were the first to examine the relationship between five firefighting events and physical performance parameters. In their study, it was indicated that firefighting tasks (stair climbing, carrying and pulling hoses, carrying or dragging a rescue dummy, using a sledged hammer) required maximal work capacity, and maximal levels for each of the physical performance measures tended to decrease with age. The authors concluded that the loss of fitness associated with advanced age is a limiting factor for the successful completion of firefighting tasks [33]. In our study, individuals aged 41 years and older required significantly more time to complete five out of the six tasks assessed. Among the younger age groups, significant differences in completion time were observed in only one of the six tasks evaluated. Based on our findings, the time needed to complete the specific firefighting tasks tended to decline only among individuals aged 41 and above. In contrast, the total time needed to complete the test differed among all the age groups. At the same time, research demonstrated that the time to complete job performance tasks is directly associated with lean muscle mass and aerobic fitness [34]. Moreover, Rhea and Colleagues (2004) demonstrated significant correlations between job-related tasks and several fitness parameters such as bench press strength, handgrip strength, bench press endurance, shoulder bench endurance, and 400 m sprint time [35]. Furthermore, research indicated that upper body muscular strength and endurance, along with a lower body fat percentage, are significantly associated with improved performance in simulated firefighting events [9,10]. 

Regarding firefighters’ performance, our results demonstrated a statistically significant difference in physical fitness tests based on age. More specifically, age had a statistically significant impact on abdominal strength, relative power during the step test, and the maximum number of push-up and sit-up repetitions performed within a minute. Finally, no significant differences in either the relative or absolute power of the vertical jump, handgrip strength, 1-RM bench press, 1-RM squat, or absolute power of the step test were indicated based on age. A similar study that examined the physical fitness of firefighters based on age indicated no changes in the 1-RM bench press and other related fitness parameters with age [36]. The authors only reported a decline in abdominal endurance with age, which agrees with our results [36]. Similar research on Canadian firefighters demonstrated that while their strength parameters remained unchanged with age, the aerobic capacity of the firefighters experienced a decline [37]. These results are comparable to ours as age did not influence vertical jump performance, handgrip strength, absolute power on the step test, or upper and lower body strength. At the same time, the number of push-up and sit-up repetitions was significantly less in older firefighters. Given that the quantity of push-ups and sit-up repetitions is directly related to the duration required to complete simulated firefighting events [10], these findings might explain the significantly poorer performance of the older group on the ability test. Additional studies provide supporting evidence that muscular endurance and cardiovascular fitness stand as the most significant predictors for completing firefighting events quickly [38]. Last but not least, a recent study has shown that older firefighters with poor body composition and reduced physical fitness levels exhibit decreased performance in all occupational tasks [38]. In the same study, the authors reported significant negative correlations between handgrip strength, upper body muscular endurance, core endurance, upper body strength, lower-body strength, and occupational tasks [39].

Furthermore, our results indicated differences in the relative but not absolute power on the step test, which is associated with the increased weight of older firefighters. Even though there were no statistical differences in weight among the different age groups, the older firefighters were heavier than the younger ones in our study. A number of longitudinal studies indicated that firefighters gain approximately 0.5 kg per year as they age, with the younger age groups gaining more weight than the older groups [4,40]. These findings suggest that weight management should be a primary concern for firefighters of all ages. Considering, however, the link between higher body fat levels and the infiltration of fat and connective tissue into muscle tissues due to muscle fiber atrophy [41,42], the maintenance of an unhealthy body fat percentage becomes particularly concerning for older firefighters.

## 5. Conclusions

The present study reinforces earlier research indicating an age-related decline in certain physical fitness parameters among firefighters. Our findings emphasize the importance of concentrating on abdominal strength and endurance alongside upper body muscular endurance. It is, therefore, advisable for firefighters to prioritize these areas in their fitness regimen to enhance occupational performance and potentially minimize the impact of age-related physical decline. In our research, strength- and power-related parameters remained relatively unchanged up to the age of 50. Additionally, firefighters should concentrate on maintaining healthy body weight and body fat, as these factors are associated with enhanced performance in firefighting tasks and have been shown to rise with age.

The results of this study yield noticeable practical applications for firefighters, fire departments, and fitness professionals, particularly in the context of optimizing performance among emergency response personnel. These findings offer a better understanding as to which fitness parameters are affected in older firefighters. Furthermore, our results can guide fitness experts and fire departments in tailoring programs to meet the unique requirements of emergency workers, as this focus is vital in fulfilling their work demands. Firefighters might need to participate in educational workshops that highlight the importance of a healthy body weight and body fat percentage while also focusing on maintaining abdominal strength and endurance, as well as upper body muscular endurance. Lastly, fire departments should promote health screenings, nutritional education programs, and exercise participation for all firefighters to maximize occupational performance and minimize the risk of health-related issues. A major limitation of this study is the lack of cardiopulmonary measurements. Hence, future studies that directly measure cardiopulmonary parameters during simulated firefighting events are encouraged. 

## Figures and Tables

**Table 1:** Anthropometric and body composition characteristics (mean ± SD).

Parameter	Age Category 20–30 Years (1)	Age Category 31–40 Years (2)	Age Category 41–50 Years (3)	Pairwise Comparisons			Effect Size
	(n = 30)	(n = 33)	(n = 17)		Mean Difference	Significance	η^2^
Age (years)	26.93 ± 2.41	34.03 ± 2.26	44.96 ± 2.93	1-2	−0.71 *	0.00	0.69
				2-3	−10.91 *	0.00	
				1-3	−18.01 *	0.00	
Weight (kg)	93.63 ± 12.83	96.74 ± 17.57	101.04 ± 14.86	1-2	−3.11	0.43	
				2-3	−4.31	0.35	
				1-3	−7.42	0.12	
Body fat (%)	20.40 ± 4.72	22.62 ± 5.61	26.13 ± 5.59	1-2	−2.22	0.10	0.14
				2-3	−3.51 *	0.03	
				1-3	−5.73 *	0.00	
BMI (kg/m^2^)	28.46 ± 2.97	29.12 ± 3.79	30.90 ± 4.19	1-2	−0.65	0.47	
				2-3	−1.79	0.10	
				1-3	−2.44 *	0.03	
Height (cm)	181.02 ± 5.42	181.82 ± 7.81	181.27 ± 6.42	1-2	−0.80	0.06	
				2-3	1.18	0.54	
				1-3	0.38	0.85	
Waist circumference (cm)	93.67 ± 11.61	98.25 ± 11.50	101.81 ± 6.42	1-2	−4.58	0.09	0.08
				2-3	−3.56	0.27	
				1-3	−8.14 *	0.02	
Hip circumference (cm)	106.06 ± 8.10	109.52 ± 7.25	107.49 ± 5.04	1-2	−3.46	0.06	
				2-3	2.03	0.35	
				1-3	−1.43	0.52	
Waist-to-hip ratio (cm)	0.88 ± 0.07	0.90 ± 0.07	0.95 ± 0.05	1-2	−0.01	0.42	0.12
				2-3	−0.05 *	0.02	
				1-3	−0.06 *	0.00	

Note: * *p* < 0.05, BF%: body fat percentage.

**Table 2:** Firefighter’s completion times of firefighting tasks (mean ± SD).

Parameter	Age Category 20–30 Years (1)	Age Category 31–40 Years (2)	Age Category 41–50 Years (3)	Pairwise Comparisons			Effect Size
	(n = 30)	(n = 37)	(n = 17)		Mean Difference	Significance	η^2^
Overall AT time (min)	6.20 ± 1.24	7.13 ± 1.49	8.45 ± 2.07	1-2	−0.93 *↑	0.02	0.22
				2-3	−1.33 *↑	0.00	
				1-3	−2.25 *↑	0.00	
Stair climb (min)	1.32 ± 0.18	1.63 ± 0.37	2.19 ± 1.36	1-2	−0.31	0.06	0.19
				2-3	−0.56 *↑	0.01	
				1-3	−0.87 *↑	0.00	
Rolled hose lift and move (min)	1.17 ± 0.27	1.34 ± 0.17	1.63 ± 0.47	1-2	−0.16 *↑	0.02	0.25
				2-3	−0.29 *↑	0.00	
				1-3	−0.46 *↑	0.00	
Keiser sled (min)	0.37 ± 0.22	0.46 ± 0.32	0.71 ± 0.43	1-2	−0.88	0.26	0.13
				2-3	−0.25 *↑	0.01	
				1-3	−0.33 *↑	0.00	
Hose pull and hydrant hookup (min)	0.74 ± 0.29	0.82 ± 0.34	0.95 ± 0.41	1-2	−0.08	0.36	
				2-3	−0.13	0.20	
				1-3	−0.21	0.06	
Rescue mannequin drag (min)	0.16 ± 0.08	0.20 ± 0.10	0.28 ± 0.22	1-2	−0.04	0.18	0.11
				2-3	−0.08 *↑	0.04	
				1-3	−0.12 *↑	0.00	
Charged hose advance (min)	0.10 ± 0.03	0.11 ± 0.03	0.13 ± 0.05	1-2	−0.01	0.09	0.11
				2-3	−0.02	0.07	
				1-3	−0.03 *↑	0.00	

Note: * *p* < 0.05, AT: ability test, ↑: increased or higher.

**Table 3:** Firefighter’s performance of physical fitness tests (mean ± SD).

Parameter	Age Category 20–30 Years (1)	Age Category 31–40 Years (2)	Age Category 41–50 Years (3)	Pairwise Comparisons			Effect Size
	(n = 30)	(n = 33)	(n = 17)		Mean Difference	Significance	η^2^
Abdominal strength (kg)	37.65 ± 11.97	34.29 ± 10.58	28.95 ± 8.68	1-2	3.35	0.22	0.08
				2-3	5.34	0.22	
				1-3	8.70 *↓	0.01	
Power vertical jump (W)	1451.75 ± 228.76	1555.55 ± 291.54	1542.56 ± 205.85	1-2	−103.80	0.11	
				2-3	12.98	0.86	
				1-3	−90.81	0.24	
Relative power—vertical jump (W/kg)	16.04 ± 2.08	15.76 ± 1.27	15.23 ± 1.42	1-2	0.29	0.50	
				2-3	0.53	0.29	
				1-3	0.81	0.11	
Left handgrip (kg)	55.22 ± 7.05	59.39 ± 8.98	57.44 ± 7.75	1-2	−4.17 *↑	0.04	
				2-3	1.94	0.42	
				1-3	−2.23	0.36	
Right handgrip (kg)	58.40 ± 6.68	62.19 ± 9.80	60.27 ± 6.46	1-2	−3.79	0.07	
				2-3	1.92	0.43	
				1-3	−1.87	0.50	
1-RM bench press (kg)	106.23 ± 20.13	98.85 ± 17.59	106.42 ± 14.16	1-2	7.38	0.11	
				2-3	−7.58	0.16	
				1-3	−0.19	0.97	
1-RM squat (kg)	136.37 ± 25.57	129.99 ± 16.69	125.41 ± 16.98	1-2	6.38	0.22	
				2-3	4.58	0.46	
				1-3	10.95	0.08	
Power—step test (W)	428.88 ± 80.01	393.48 ± 101.46	401.64 ± 99.04	1-2	35.40	0.15	
				2-3	24.05	0.41	
				1-3	59.44 *↓	0.05	
Relative power—step test (W/kg)	4.81 ± 1.14	4.20 ± 1.15	3.63 ± 1.03	1-2	0.61 *↓	0.03	0.14
				2-3	0.57	0.10	
				1-3	1.18 *↓	0.00	
Push-ups (max reps)	42.93 ± 16.99	33.82 ± 15.60	29.53 ± 9.49	1-2	9.12 *↓	0.02	0.12
				2-3	4.29	0.35	
				1-3	13.40 *↓	0.00	
Sit-ups (reps in a minute)	41.40 ± 12.61	37.64 ± 9.71	30.41 ± 9.96	1-2	3.76	0.18	0.13
				2-3	7.22 *↓	0.03	
				1-3	10.99 *↓	0.00	

Note: * *p* < 0.05, 1-RM:1 repetition max, ↓: decreased or lower; ↑: increased or higher.

## Data Availability

The data presented in this study are available upon request from the corresponding author.
